# Female Physicians in Earlier Times

**Published:** 1890-12

**Authors:** 


### Female Physicians in Earlier Times.

In 1596 a license was granted in the city of Norwich, England, to Dame Adrian Colman, widow, late wife of Dr. Nicolas Colman. The license was given this lady to administer to women and children on the grounds of her having been experienced in the art of physic during the lifetime of the said Nicolas. In 1560 the Bishop's Court of Norwich licensed Dame Cecily Baldrye to practice the art of chirurgery. One hundred years later a dentist, Robert Purland, received a permit to practice from the bishop of the same diocese. This is cited to show the scope of power of the ecclesiastics of those early times. It is not generally known that in the early Middle Ages much of the military surgery of that period fell into the hands of women who were not unskilled in simple cures. In the old troubadour song about Aucassin and Nicolette, we are told that when the hero got his hurt, Nicolette looked him over and found his arm out of joint and " put it in again." In the feudal times it was the custom to educate the girls, belonging to the nobles, in practical matters pertaining to medicine and surgery, especially the healing of wounds. But by the time of the sixteenth century the sphere of women, at least in the German army, had fallen off several points from the dignity of the original treatment of the wounded to a compound of nurse, camp-follower, and some thing even less desirable, which is best expressed by the title of the official who had charge of them, namely, " hurenwaibel," or whoresergeant. In camp the sick and wounded were collected into a special hospital tent and nursed by these ladies. On long marches they were carried along in wagons, or left behind in villages or cities under the care of a " spittelmeister."

---

Solvents for Camphor.—According to Sazeau, the addition to camphor of ether or alcohol increases the quantity which chloroform is capable of dissolving. To this Hoffer adds that olive oil saturated with camphor will dissolve six times as much as iodoform.—*Pharm. Journal*

